# Rebuilding the myocardial microenvironment to enhance mesenchymal stem cells-mediated regeneration in ischemic heart disease

**DOI:** 10.3389/fbioe.2024.1468833

**Published:** 2024-09-20

**Authors:** Qing Chu, Xin Jiang, Ying Xiao

**Affiliations:** ^1^ Regenerative Medicine Research Center, Sichuan University West China Hospital, Chengdu, Sichuan, China; ^2^ Department of Laboratory Medicine, Sichuan University West China Hospital, Chengdu, Sichuan, China; ^3^ Innovation Institute for Integration of Medicine and Engineering, Sichuan University West China Hospital, Chengdu, Sichuan, China; ^4^ Department of Postgraduate, Sichuan University West China Hospital, Chengdu, Sichuan, China

**Keywords:** mesenchymal stem cells, microenvironment, agiogenesis, myocardial regeneration, ischemic heart disease

## Abstract

Mesenchymal stem cells (MSCs) are naturally-derived regenerative materials that exhibit significant potential in regenerative medicine. Previous studies have demonstrated that MSCs-based therapy can improve heart function in ischemia-injured hearts, offering an exciting therapeutic intervention for myocardial ischemic infarction, a leading cause of worldwide mortality and disability. However, the efficacy of MSCs-based therapies is significantly disturbed by the myocardial microenvironment, which undergoes substantial changes following ischemic injury. After the ischemic injury, blood vessels become obstructed and damaged, and cardiomyocytes experience ischemic conditions. This activates the hypoxia-induced factor 1 (HIF-1) pathway, leading to the rapid production of several cytokines and chemokines, including vascular endothelial growth factor (VEGF) and stromal-derived factor 1 (SDF-1), which are crucial for angiogenesis, cell migration, and tissue repair, but it is not sustainable. MSCs respond to these cytokines and chemokines by homing to the injured site and participating in myocardial regeneration. However, the deteriorated microenvironment in the injured myocardium poses challenges for cell survival, interacting with MSCs, and constraining their homing, retention, and migration capabilities, thereby limiting their regenerative potential. This review discusses how the deteriorated microenvironment negatively affects the ability of MSCs to promote myocardial regeneration. Recent studies have shown that optimizing the microenvironment through the promotion of angiogenesis can significantly enhance the efficacy of MSCs in treating myocardial infarction. This approach harnesses the full therapeutic potential of MSCs-based therapies for ischemic heart disease.

## 1 Introduction

Ischemic heart disease (IHD), caused by stenosis or blockage of the coronary arteries resulting in a lack of blood supply to the myocardium, remains one of the leading causes of death worldwide (Bradley and Berry, 2022; Pastena et al., 2024). Following myocardial ischemia, there is an extensive loss of cardiomyocytes, which are then replaced by excessive collagen deposition. This leads to impaired heart contraction and relaxation, eventually resulting in heart failure (Stone et al., 2023). Current clinical treatments, such as vasodilation, diuresis, and inotropic therapies, can temporarily relieve ischemic symptoms but do not regenerate new cardiomyocytes for functional recovery (Ahmadi et al., 2016; Boden et al., 2023; Niccoli et al., 2021). Given the limited self-renewal capacity of cardiomyocytes, cell-based strategies to replenish lost cardiomyocytes or promote endogenous repair offer a new option for patients (Hsiao et al., 2013; Madonna, 2022).

Mesenchymal stem cells (MSCs) are naturally derived regenerative materials with the capacity for self-renewal and multi-lineage differentiation, and they are widely distributed across various tissues (Castilla-Casadiego et al., 2020; Chang et al., 2021; Cherian et al., 2020). MSCs-based therapies have shown promising potential in promoting myocardial recovery from ischemic injury (Ward et al., 2018). However, the mechanistic understanding of MSCs in myocardial regeneration remains controversial (Najar et al., 2021). Although a growing body of preclinical and clinical studies has observed improvements in heart function, more cautious analyses have revealed that the quantity of cardiomyocytes differentiated from MSCs is far below the sufficiency needed to support significant myocardial recovery from MSCs treatment (Ward et al., 2018; Eschenhagen et al., 2017).

One of the most significant challenges is the low efficiency of MSCs homing to the infarcted heart, with cells often redistributing to other organs, including the lung, liver, and spleen (Chan et al., 2022; Penna et al., 2008). Additionally, a rapid loss of transplanted MSCs occurs within the first 48 h after transplantation via intravenous, intracoronary, or intramyocardial injection (Malik et al., 2020; Chin et al., 2024). Although there are techniques to enhance MSCs retention at the injured myocardium, such as cell patches, these MSCs primarily exert their effects through paracrine mechanisms, improving the microenvironment by promoting angiogenesis, reducing fibrosis, and modulating inflammation (Shi et al., 2021; Wu and Zhao, 2012; Yan et al., 2022; Zhang et al., 2016a). These findings suggest a critical issue: the ischemia-injured myocardium is unsuitable for MSCs homing, retention, and differentiation into cardiomyocytes, thereby limiting their potential for myogenesis.

The myocardial microenvironment is composed of various physiological, chemical, and biological factors primarily generated by non-cardiomyocyte cells, including immune cells, stromal cells, and vascular cells (Tang et al., 2020). Following ischemic injury, these cells undergo substantial changes, leading to a dynamic shift in the microenvironment (Li et al., 2021). Immune cells such as macrophages, neutrophils, and lymphocytes are rapidly activated in response to ischemic insult, migrating to the injured myocardium to eliminate debris from dead cells (Sun et al., 2021). The vasculature is damaged due to the ischemia-induced loss of vascular cells, including endothelial cells, pericytes, and smooth muscle cells (Lupu et al., 2020). Angiogenesis is then initiated, relying on viable endothelial cells from collateral vessels, contributing to the reconstruction of vasculature and partially alleviating hypoxia (Xiao et al., 2020). Stromal cells, mainly fibroblasts, are activated and transformed into myofibroblasts, producing abundant collagen and facilitating collagen crosslinking, leading to cardiac fibrosis (Li et al., 2014). These changes significantly impact the biological activities and functions of MSCs in myocardial regeneration (Khalil and McCain, 2021).

In this review, we summarized the current understanding of MSCs involved in myocardial regeneration and the relationship between changes in the myocardial microenvironment and the biological activities of MSCs during ischemic injury progression, providing a novel insight into the critical role of rebuilding the microenvironment in promoting the efficacy of MSCs in myocardial regeneration.

## 2 MSCs applied in IHDs therapy

MSCs are multipotent stem cells characterized by self-renewal, robust proliferative capacity, and multilineage differentiation potential. MSCs predominantly express positive markers such as CD29, CD90, CD105, and CD44, while showing negative expression of hematopoietic and vascular markers like CD45, CD34, CD19, CD11b, and CD14 (Dominici et al., 2006). MSCs can differentiate into various mesoderm lineages and cell types, including osteoblasts, adipocytes (Casado-Diaz et al., 2016), skeletal muscle myocytes/myotubes (Park et al., 2016), and cardiomyocytes (Makino et al., 1999) under growth factor-rich selective media. MSCs can be derived from a wide range of sources, including bone marrow, adipose tissue, umbilical cord, placenta, and dental pulp (Prakash et al., 2023). Bone marrow-derived MSCs (BM-MSCs) were the first identified and isolated from bone marrow (Friedenstein et al., 1970; Friedenstein et al., 1966) and have emerged as one of the leading candidates for clinical translational applications. Due to the invasive nature of harvesting BM-MSCs and their poor cell viability, alternative sources of MSCs have been explored. Among these, umbilical cord mesenchymal stem cells (UC-MSCs) are considered one of the most ideal sources for transplantation therapy due to their ease of collection, wide availability, and low immunogenicity (Sriramulu et al., 2018). Additionally, adipose-derived mesenchymal stem cells (AD-MSCs) exhibit stronger immunomodulatory properties compared to other MSCs and have also been extensively studied (Czerwiec et al., 2023). While MSCs from different sources have varying characteristics in terms of collection, proliferation, differentiation, and functional regulation (Hoogduijn et al., 2014), their capacity for myocardial regeneration is recognized for its encouraging potential in the therapeutic effect of IHD. Since the early 2000s, when landmark studies reported that bone marrow cells could potentially replace damaged myocardium in the adult heart (Orlic et al., 2001), MSCs have been studied for the treatment of IHD for over 20 years, yielding promising preclinical results and mixed clinical outcomes.

### 2.1 Preclinical studies

Since Orlic et al. reported the potential of bone marrow cells to replace damaged adult myocardium in 2001, a finding that was later challenged, MSCs-based therapies for myocardial regeneration have been extensively studied. These studies have utilized differentiated or undifferentiated MSCs from allogeneic, autologous, and even xenogeneic sources, employing various delivery approaches (Huang et al., 2010; Jansen Of Lorkeers et al., 2015; Luger et al., 2017; Tomita et al., 1999). Meta-analyses have reported an overall 12% increase in left ventricular ejection fraction (LVEF) in rodent studies following MSCs administration compared to untreated groups. Additionally, a 7% increase in LVEF was observed in large species such as pigs, with only 7 out of 16 studies showing favorable results for MSCs. Correspondingly, an 8% reduction in infarct size was observed in half of the preclinical studies in rodents, and a 6.4% reduction in pig hearts was noted in the seven studies that reported favorable results for MSCs administration (Kanelidis et al., 2017).

### 2.2 Clinical trials

Most clinical trials of cell therapy for IHD have concentrated on BM-, with AD- and UC-MSCs also being studied in recent years (Ward et al., 2018). These trials have demonstrated favorable safety and tolerability. The treatment of acute myocardial infarction (AMI) typically involves the intracoronary injection of MSCs following percutaneous coronary intervention (PCI), with intravenous injection being used in some cases. A meta-analysis of 13 clinical trials in AMI reported a highly significant 3.78% increase in LVEF for the MSCs-treated group compared to the control group (Attar et al., 2021). MSCs have also been investigated for the treatment of chronic ischemia and ischemic cardiomyopathy. The primary route of treatment for chronic myocardial infarction (MI) is intramyocardial, rather than intracoronary or intravenous (Wang et al., 2015a). A meta-analysis demonstrated the efficacy of MSCs, highlighting the overall safety and efficacy of autologous BM-MSCs in chronic MI treatment (Kanelidis et al., 2017). A randomized controlled trial by Zhang et al. assessed the efficacy of MSCs transplantation in patients with chronic MI, reporting significant improvements in LVEF and reduced infarct size (Wang et al., 2015b). These findings supported the long-term outcomes of MSCs therapy, observing improved survival rates, reduced rehospitalization, and enhanced quality of life among treated patients (Afzal et al., 2015). Nowadays, there are 2 related MSC products approved for the treatment of peripheral vascular disease. One is Stempeucel for atherosclerotic and non-atherosclerotic critical limb ischemia, developed by Stempeutics; and the other is Vescell (ACP-01), under development by Hemostemix, for the treatment of IHD. Several clinical trials are currently underway for the treatment of IHD using UC-MSCs (Table 1).

**TABLE 1:** Summary of the ongoing clinical trials for IHD.

Trial ID	Trial title	Cell source	Condition	Phase	Routine
NCT06147986	Evaluate the Efficacy and Safety of Allogeneic Umbilical Cord Mesenchymal Stem Cells as an Add-On Treatment for Acute ST-elevation Myocardial Infarction (STEMI) Patients	Allogeneic umbilical cord mesenchymal stem cells	ST-elevation Myocardial Infarction	III	Intracoronary and Intravenous
NCT05935423	Umbilical Cord Mesenchymal Stem Cell Improve Cardiac Function on ST-elevation Myocardial Infarction (STEMI) Patients	Umbilical Cord Mesenchymal Stem Cell	ST Elevation Myocardial Infarction	III	Intracoronary
NCT05043610	MSCs for Prevention of MI-induced HF	Umbilical Cord Mesenchymal Stem Cell	Myocardial Infarction, Acute	III	Intracoronary
NCT04776239	Allogeneic Mesenchymal Human Stem Cell Infusion Therapy for Endothelial DySfunctiOn in Diabetic Subjects With Symptomatic Ischemic Heart Disease. (ACESO-IHD)	Umbilical Cord Mesenchymal Stem Cell	Ischemic Heart Disease; Diabetes Mellitus	I/II	Intravenous

### 2.3 Mechanisms of MSCs in promoting myocardial regeneration

#### 2.3.1 Differentiation, paracrine effects and immunomodulation of MSCs

Numerous studies have demonstrated the ability of MSCs to differentiate into cardiomyocytes, endothelial cells, and smooth muscle cells. Notably, evidence of MSCs differentiating into functional cardiomyocytes, including the acquisition of contractile properties and the expression of cardiomyocyte marker genes *in vitro*, has been observed after treatment with 5-azacytidine, a hypomethylating agent (Tomita et al., 1999). Due to safety concerns, further studies have shifted from the use of 5-azacytidine to alternative agents, such as insulin and dexamethasone, to induce MSCs differentiation into cardiomyocytes (Shim et al., 2004). Transplantation of these MSCs-derived cardiomyocytes into ischemic-injured hearts has been shown to contribute to myocardial functional recovery (Iso et al., 2007). Of note, undesirable integration of these transplanted cells with the resident cardiomyocytes, and the occurrence of arrhythmia post-transplantation primarily hinders the application of MSCs in the treatment of IHD (Yagyu et al., 2019). Moreover, several studies have reported when MSCs were directly transplanted into the myocardium, they rarely differentiated into cardiomyocytes, possibly ascribed to lacking of appropriate signals and microenvironment (Eschenhagen et al., 2017; Leiker et al., 2008). Results from animal and patients have demonstrated that MSCs can improve cardiac function, although this improvement is likely not due solely to the replacement of injured contractile cardiomyocytes. With the advancement of research technology, more evidence shows that MSCs promote the improvement of cardiac function not through the differentiation into cardiomyocytes but through paracrine effects and immunomodulation (Gallina et al., 2015).

Paracrine effects are currently widely studied for understanding MSCs-induced myocardial regeneration (Li et al., 2023). MSCs primarily secrete various growth factors and cytokines, such as vascular endothelial growth factor (VEGF), fibroblast growth factor (FGF), and insulin-like growth factor-1 (IGF-1) (Gallina et al., 2015). These factors promote angiogenesis in ischemic regions, improving blood supply to the myocardial tissue and providing necessary nutrients and oxygen for cardiomyocyte regeneration (Yin et al., 2023). Additionally, MSCs produce anti-fibrotic factors that reduce scar tissue formation by inhibiting fibroblast-to-myofibroblast transition and reducing collagen fiber deposition (Takahashi et al., 2006). Moreover, MSCs secrete immunomodulatory factors that inhibit T-cell activation, thereby reducing the production of pro-inflammatory cytokines that might further damage cardiomyocytes (Gomez-Ferrer et al., 2021).

MSCs can activate intrinsic immune responses. Direct injection of adult stem cells can trigger an acute inflammatory response characterized by CCR2^+^ and CX3CR1^+^ macrophage accumulation, which alters fibroblast activity, reduces fibrosis, and enhances the mechanical properties of the injured heart (Vagnozzi et al., 2020). Furthermore, MSCs interact directly with immune cells, such as macrophages, T-cells, and natural killer (NK) cells. They can polarize macrophages towards an M2 phenotype, associated with anti-inflammatory and pro-repair activities. This shift promotes the clearance of cellular debris and supports angiogenesis while inhibiting the release of harmful pro-inflammatory cytokines (Ben-Mordechai et al., 2013). Similarly, MSCs can suppress T-cell proliferation and activation, reducing the risk of allograft rejection and alloreactivity in transplantation settings (Martinez et al., 2017).

#### 2.3.2 The processes of MSCs participation in myocardial regeneration

The underlying processes of MSCs in myocardial regeneration involve their homing to the injured myocardium and retention there for subsequent functions. These processes require precise regulation of multiple signals and structural support.

##### 2.3.2.1 Homing

MSCs homing refers to the biological activities that enable MSCs to move towards the injured site, which is the first and foremost step for their participation in myocardial regeneration (Szydlak, 2019). The direction of MSCs migration is determined by several cytokines, chemokines, and pro-inflammatory factors. These signals, produced in response to ischemic injury, act as navigators for MSCs homing and are termed tissue injury signals. Stromal cell-derived factor-1 (SDF-1), also known as CXCL12, is one of the most important tissue injury signals for MSCs homing (Zhang et al., 2016a). After ischemic injury, SDF-1 proteins are rapidly upregulated in the injured myocardium and released into the circulation (Rota, 2010). SDF-1 proteins bind to the receptor CXCR4 on MSCs, activating intracellular signal transduction via the mitogen-activated protein kinase (MAPK) pathways to phosphorylate cytoskeletal proteins like vimentin for cell migration (Chen et al., 2020; Tang et al., 2011). Consequently, MSCs are mobilized from their resident niche into the circulation and then move towards the injured myocardium (Tang et al., 2011). Other tissue injury signals, including pro-inflammatory factors (interleukins [ILs] and tumor-necrosis factor-α [TNF-α]) and growth factors (VEGF, PDGF and TGF-β), also play a role in MSCs homing (Szydlak, 2019). The process of MSCs homing generally occurs in the early phase after ischemic injury, accompanied by the upregulation of tissue injury signals at the injured site (Kang and Zheng, 2013).

The conduit for tissue injury signal transduction is primarily the vasculature-dependent circulation system. This conduit is essential for the movement of circulating stem cells and their arrival at the injury site (Kang and Zheng, 2013). When MSCs reach the injured site, regional chemokines and cytokines in high concentrations bind to receptors on the MSCs, mediating their rolling along endothelial cells. Thereafter, adhesive molecules like vascular cell adhesion molecule-1 (VCAM-1) on the surface of endothelial cells bind to integrins on the membranes of MSCs, inducing MSCs adhesion and subsequent transmigration through the endothelial layer, thus completing MSCs homing (Segers et al., 2006; Nitzsche et al., 2017).

Direct injection of MSCs into the injured myocardium bypasses the homing process, and forces MSCs to remain there. This technique is widely used in MSC-based therapy for IHDs but is an invasive procedure that brings additional risks to patients (Kanelidis et al., 2017). Moreover, intramyocardial injection of MSCs can create isolated cell islands with poor connections to native cardiomyocytes, potentially causing arrhythmias in the future (Yagyu et al., 2019).

##### 2.3.2.2 Retention

After homing to the injured myocardium, MSCs must survive and be colonized there for long-term retention. Essential oxygen and nutrients are necessary for the survival of MSCs, and both are mainly provided by vasculature. Some research has demonstrated that MSCs can tolerate lower oxygen levels (5%) via the activation of the HIF-1 signaling pathway, with their paracrine function to promote angiogenesis being enhanced under hypoxic conditions compared to normal conditions with 21% oxygen (Sun et al., 2020a). However, in the context of MSCs transplantation in IHDs, deprivation of oxygen and nutrients by damaged vasculature usually creates a deteriorated microenvironment, leading to massive MSCs apoptosis post-transplantation (Chen et al., 2018). Poor retention of transplanted MSCs is a significant drawback that limits the clinical application of MSCs-based therapy for IHDs (Kanki et al., 2011).

The colonization of MSCs after homing to the injured myocardium is also regulated by mechanical stress provided by the extracellular matrix (ECM) (Raimondi et al., 2013). MSCs are sensitive to changes in mechanical stress via ion channels on the cell surface, including Piezo1, which activates the intracellular Hippo pathway effector YAP/TAZ to regulate cell morphology, proliferation, and differentiation. A previous study revealed that a 3D soft and elastic hydrogel could effectively enhance cell growth, proliferation, and osteogenic differentiation of MSCs (Di et al., 2023). Additionally, the differentiation of embryonic stem cells (ESCs) into cardiomyocytes favored 3D cultures with less mechanical stress than 2D cultures (Ou et al., 2011). However, an increase in mechanical stress due to excessive collagen deposition after ischemic injury may hinder the colonization, proliferation, and differentiation of transplanted MSCs (Li et al., 2014).

##### 2.3.2.3 Promotion of myocardial regeneration

After MSCs homing and retention in the injured myocardium, they can perform functions such as secretion and differentiation for myocardial regeneration.

The role of MSCs in myocardial regeneration is debated, particularly whether they can differentiate into cardiomyocytes to replace lost cardiomyocytes after ischemic injury, as we also noted above. One crucial requirement for MSCs differentiation is the induction of specific signals (Eschenhagen et al., 2017). During heart development, several factors, including BMPs, WNTs, and FGFs, and the sequential activation of transcription factors like MIXL1, Nkx2.5, and GATA4, are involved in the differentiation from MSCs into cardiomyocytes (Buijtendijk et al., 2020). The first report in 1999 indicated the induction of adult MSCs differentiation into cardiomyocyte-like cells with sarcomere for spontaneous contraction by adding 5-azacytidine into the cultures (Makino et al., 1999; Tomita et al., 1999). Further studies have developed various methods by combining different chemicals to improve the efficiency of MSCs differentiation into cardiomyocytes, and these induced cardiomyocytes have been applied for cell transplantation in IHDs for myocardial regeneration (Shim et al., 2004). However, little studies have observed that MSCs differentiate into cardiomyocytes *in vivo* (Leiker et al., 2008), likely due to the lack of appropriate signals in the microenvironment.

## 3 Interplay between MSCs and the altered microenvironment after ischemic injury

The myocardial microenvironment is a complex system composed of various non-cardiomyocytes, including immune cells, vascular cells, and fibroblasts, as well as the non-cellular extracellular matrix. It plays a critical role in modulating the behavior of MSCs and influencing their ability to effectively participate in tissue repair and regeneration (Franchi et al., 2020).

### 3.1 Pathological changes after myocardial ischemia

**FIGURE 1:**
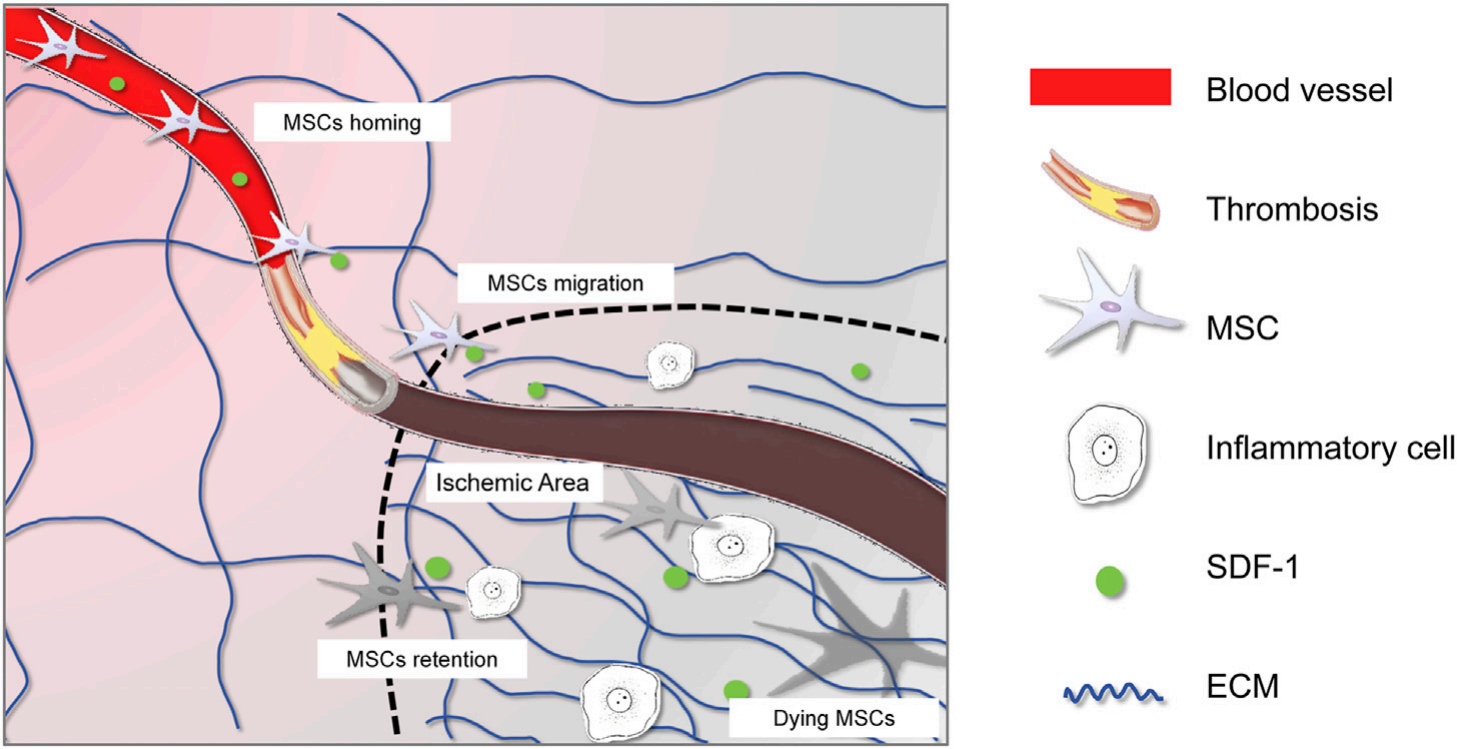
Interplay between MSCs and the altered microenvironment after ischemic injury. The graphic symbols were listed in the right panel.

The dramatic pathological changes following myocardial ischemia include the massive loss of cardiomyocytes, destruction of microvessels, and recruitment and activation of immune cells (Figure 1). Current knowledge indicates that the regenerative capacity of cardiomyocytes is extremely weak, leading to fibrotic repair to maintain the heart’s integrity. Various fibrotic mediators and cytokines released by macrophages, lymphocytes, and other cells create a fibrotic microenvironment in the ischemic area, stimulating the transformation of fibroblasts into myofibroblasts (Xiao et al., 2023). These myofibroblasts produce large amounts of extracellular matrix proteins, leading to collagen deposition and cardiac fibrosis, which induces adverse remodeling that can gradually progress to heart failure (Prabhu and Frangogiannis, 2016).

### 3.2 Change of vasculature and the deteriorated microenvironment

Appropriate vasculature is crucial for the survival and normal function of cardiac cells, as it provides essential oxygen, nutrients, growth factors, and cytokines (Brutsaert, 2003). Ischemic injury, arising from the obstruction of blood vessels, can lead to further structural damage in these vessels (Xiao et al., 2021a). Endothelial cells, in particular, are highly susceptible to ischemic insults and may experience impaired integrity and increased permeability. This disruption of the endothelial barrier facilitates the leakage of fluid and macromolecules into the surrounding tissue, exacerbating the ischemic injury (Chu et al., 2023). Additionally, ischemic insults can damage the basement membrane, resulting in the loss of its structural support and regulatory functions. Furthermore, smooth muscle cells in the vessel wall may undergo apoptosis or dysfunction, compromising the structural integrity of blood vessels and contributing to the progression of vascular remodeling and dysfunction (Heitzer et al., 2001). In response to ischemia-induced hypoxia, angiogenesis is initiated, sprouting from existing endothelial cells and expanding towards the injured site to recover blood supply (Nofi et al., 2018). However, angiogenesis is often not sustained, and vascular remodeling further impairs the ability of blood vessels to withstand hemodynamic stress (Xiao et al., 2021a).

The ischemic injury leading to vascular changes directly affect the survival and migration of MSCs. As blood vessels are the primary route for MSCs to obtain oxygen, nutrients, and growth factors, vascular obstruction or damage disrupts the microenvironment, compromising their survival and normal functions (Kang and Zheng, 2013). On the other hand, endothelial cells losing their integrity and increasing vascular permeability might facilitate the migration of MSCs from blood vessels to the injured site (Chu et al., 2023). Additionally, in response to ischemia-induced hypoxia, MSCs might activate their paracrine effects on immunomodulation and suppress inflammatory responses (Xiao et al., 2021b). Some studies showed that hypoxia might shift the differentiation preference of MSCs towards vascular repair-related cell types, including endothelial cells and smooth muscle cells (Tian et al., 2022).

Following ischemic injury, the heart undergoes a series of intricate immune responses involving the activation and infiltration of various immune cells. These include both innate immune cells, such as neutrophils and macrophages, and adaptive immune cells, including T and B cells (Nahrendorf et al., 2007; Frangogiannis, 2014). These immune cells accumulate at the site of cardiac injury and release a multitude of inflammatory mediators and cytokines, such as interleukins (IL-1, IL-6, IL-17), tumor necrosis factor-alpha (TNF-α), chemokines (CXCL1, CCL2), and reactive oxygen species (ROS), which further promote the inflammatory response and tissue repair (Swirski and Nahrendorf, 2013). The immune response typically peaks within 4–7 days following ischemic injury and subsequently reduces to a stable state over time (Xiao et al., 2021a).

MSCs respond to cytokines and chemokines released from the injured myocardium, rapidly mobilizing and migrating towards the site of injury under the guidance of these factors (Kang and Zheng, 2013). Once in the injured myocardium, MSCs interact with various immune cells. Macrophages play a pivotal role in the inflammatory response following ischemic heart injury, with the ability to polarize into M1 (pro-inflammatory) or M2 (anti-inflammatory) phenotypes, each exerting distinct effects on MSCs (Ben-Mordechai et al., 2013). M1 macrophages release pro-inflammatory cytokines that can compromise MSCs survival and function, while M2 macrophages foster a more conducive environment for MSCs-mediated tissue repair. Reciprocally, MSCs can influence macrophage polarization, favoring the M2 phenotype, which aids in reducing inflammation and promoting healing (Neupane et al., 2023). Additionally, MSCs can mitigate the inflammatory response by suppressing T cell proliferation and activation (Behm et al., 2024), as well as inhibiting NK cell activation and cytotoxicity, thereby protecting heart tissue from immune-mediated damage. The interplay between MSCs and immune cells is dynamic, with MSCs promoting a more balanced immune response that supports tissue repair (Najar et al., 2010).

On the other hand, the ECM forms the native cellular support network and has a strong interplay with its residing cells. Following myocardial ischemic injury, the ECM undergoes significant remodeling, particularly in its composition (Chu et al., 2021). Activated cardiac fibroblasts proliferate and increase the synthesis of collagenous proteins, primarily types I and III collagen, which are then deposited in the ECM. This excessive deposition of collagenous proteins leads to fibrosis, altering the structural integrity of the myocardium (Xiao et al., 2023). In addition to collagen, non-collagenous components of the ECM, such as glycosaminoglycans, proteoglycans, elastin, and laminin, may also undergo changes in their content and distribution, further contributing to ECM remodeling (Bonnans et al., 2014).

This remodeling process creates a new microenvironment that supports MSCs migration and affects their biological behavior. Firstly, the remodeled ECM provides a structural framework and essential cues for MSCs migration. Specific signals and adhesion molecules, including growth factors, cytokines, and chemokines embedded within the remodeled ECM, could guide MSCs to the injured area, acting as chemoattractants for efficient homing and tissue repair processes (Zhu et al., 2006). Additionally, the mechanical stress altered by the remodeled ECM might change MSCs biological functions, including their paracrine effects to attenuate myofibroblast transition-induced fibrosis (Galie and Stegemann, 2014) and promote angiogenesis (Piao et al., 2005). Furthermore, MSCs could acquire contraction ability when exposed to mechanical stretch, contributing to functional recovery after ischemic injury (Choi et al., 2017; Girao-Silva et al., 2014; Izadpanah et al., 2022). However, excessive collagen deposition leads to fibrotic scar formation, resulting in a deteriorated microenvironment for MSCs characterized by stiff ECM, intensive mechanical stress, and reduced blood vessels (Li et al., 2021). Therefore, this situation should be avoided when utilizing MSCs treatment for IHDs.

## 4 Rebuilding of myocardial microenvironment for MSCs promotion of myocardial regeneration

As discussed earlier, the efficacy of MSCs-based therapies largely depends on the quality of the recipient myocardial microenvironment. Enhancing the myocardial microenvironment is pivotal for maximizing the regenerative capacity of MSCs. By addressing the underlying deficiencies in the myocardial microenvironment and creating a more favorable setting for MSCs engraftment, the regenerative potential of MSCs can be fully realized, opening new therapeutic avenues for myocardial regeneration.

**FIGURE 2:**
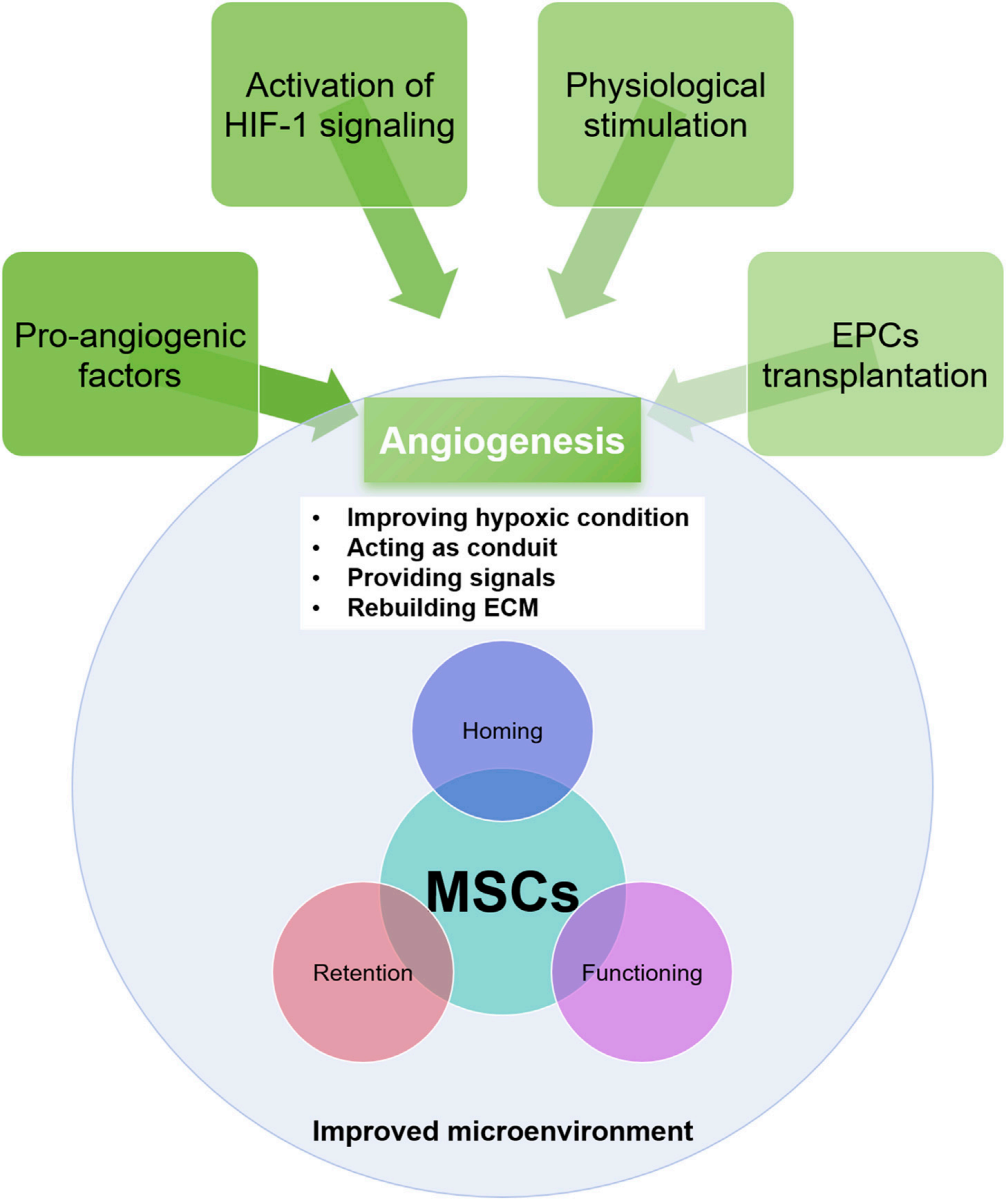
The approaches for angiogenesis and its role in rebuilding the microenvironment and in improving MSCs efficacy.

The essential role of vasculature in facilitating MSCs homing to injured tissue is well-documented (Shi et al., 2021). In IHDs, the destruction of blood vessels disrupts the signaling between the injured myocardium and MSCs and impedes MSCs migration to the injury site, ultimately hindering tissue regeneration. Numerous studies have demonstrated that promoting angiogenesis beforehand benefits the survival of transplanted MSCs (Shi et al., 2021; Chin et al., 2021). Recovering vasculature in the injured myocardium enhances the survival prospects of grafted MSCs, making angiogenesis a promising therapeutic approach (Figure 2).

### 4.1 Regulation of angiogenesis in the ischemic myocardium

The HIF-1 signaling pathway is a central regulator of angiogenesis, orchestrating the expression of multiple pro-angiogenic factors and metabolic adaptations essential for vessel formation (Semenza, 2014). HIF-1 is a heterodimeric transcription factor with two subunits: HIF-1α and HIF-1β (ARNT). HIF-1α, the oxygen-sensitive subunit, is tightly regulated by cellular oxygen levels. Under hypoxic conditions, HIF-1α escapes hydroxylation and proteasomal degradation, translocates to the nucleus, and dimerizes with HIF-1β to form an active transcription complex (Wang et al., 1995). This complex binds to hypoxia-responsive elements (HREs) in target gene promoters, inducing their expression. One crucial target of HIF-1 in angiogenesis is VEGF, which promotes endothelial cell proliferation, migration, and tube formation (Liu et al., 2018; Cheng et al., 2016). HIF-1 directly upregulates VEGF expression, initiating and enhancing the angiogenic response and revascularization to improve tissue perfusion (Liu et al., 2018; Li et al., 2012).

However, the angiogenic response following myocardial ischemic injury is often impaired (Zhang et al., 2016b). While angiogenesis is rapidly activated in response to ischemic injury, peaking at day 4 and declining after 1 week, HIF-1α consistently accumulates in the ischemic myocardium (Xiao et al., 2020; Xiao et al., 2021a). The primary issue may be impaired activation of HIF-1 regulation of angiogenic factors, leading to an imbalance between angiogenic factors and their inhibitors. This imbalance hinders new blood vessel formation and limits tissue revascularization, ultimately affecting MSCs-mediated tissue repair. Additionally, the inflammatory response following myocardial ischemia releases pro-inflammatory cytokines that inhibit angiogenesis and promote fibrosis (Gomez-Ferrer et al., 2021).

### 4.2 Rebuilding of the microenvironment and enhancement of MSCs efficacy

Several mechanisms underlie the beneficial effects of angiogenesis on the ischemic myocardial microenvironment and MSCs function (Shi et al., 2021). Firstly, new blood vessel formation increases oxygen and nutrient delivery to the injured tissue and removes waste products, creating a more conducive environment for MSCs survival and proliferation (Sun et al., 2020b). Secondly, angiogenesis stimulates the release of growth factors and cytokines beneficial for MSCs function. For instance, VEGF and FGF, potent angiogenic factors, also promote MSCs proliferation, migration, and differentiation (King et al., 2021). The restoration of functional vasculature in the ischemic myocardium provides a route for MSCs to migrate to the injury site and plays a key role in ECM reconstruction and inflammatory response modulation (Gomez-Ferrer et al., 2021; Sun et al., 2020b). Vasculature-derived ECM supports MSCs adhesion and migration, essential for efficient recruitment to damaged tissue (King et al., 2021). Angiogenesis also modulates the inflammatory response by promoting anti-inflammatory cell infiltration and reducing pro-inflammatory cytokine levels, creating a less hostile microenvironment for MSCs, allowing for better cell survival and function (Chin et al., 2021).

Promoting angiogenesis in ischemic heart disease can be achieved through activating angiogenic factors (Lupu et al., 2020). Direct intravenous injection, intramyocardial injection, or gene therapy with VEGF and FGF can significantly increase capillary density (Henning, 2016). Overexpression of HIF-1α via adenoviral or lentiviral vectors stimulates angiogenic factor expression (Huang et al., 2014). A recent study showed that while HIF-1α proteins consistently accumulate in the infarct zone, angiogenic factor expression is impaired, possibly due to dysfunction in selective HIF-1 signaling regulation. Supplementing the trace element copper can retune this dysregulation and reactivate HIF-1 target angiogenic factor expression without excessively increasing HIF-1α accumulation (Xiao et al., 2023; Zhang et al., 2016b).

Other techniques, including physical stimuli like low-intensity pulsed ultrasound (LIPUS) and electrical stimulation, have also been explored as non-invasive means to enhance VEGF expression and promote capillary formation (Amaral et al., 2001; Li et al., 2022). Electrical stimulation stimulates the release of angiogenic growth factors from endogenous cells and promotes neovascularization (Zhao et al., 2021). Both techniques have shown positive effects on angiogenesis and cardiac function in preclinical studies.

Another approach involves cell-based therapies, particularly using endothelial progenitor cells (EPCs). EPCs exhibit inherent angiogenic properties and secrete a diverse range of growth factors promoting blood vessel formation. When injected into the ischemic myocardium, EPCs can differentiate into vascular endothelial cells, directly participating in new blood vessel formation (Ghem et al., 2017; Zhang et al., 2008). Combining MSCs and EPCs for ischemic heart disease treatment represents an innovative and promising therapeutic approach. MSCs, known for their immunomodulatory effects, reduce inflammation and promote tissue repair, while EPCs specifically target endothelial cells regeneration. This combination harnesses the regenerative and angiogenic potential of these stem cells to promote cardiac tissue repair and restore blood flow to ischemic regions of the heart.

## 5 Conclusion and perspective

In conclusion, MSCs have emerged as a promising therapeutic option for IHDs due to their paracrine properties, which promote angiogenesis, modulate inflammatory responses, and inhibit the fibrotic process. However, the efficacy of MSCs-based therapies is significantly disturbed by the myocardial microenvironment, which undergoes dramatic changes following ischemic injury. Rebuilding the myocardial microenvironment, particularly by promoting angiogenesis, is a pivotal strategy to enhance the regenerative capacity of MSCs. Angiogenesis improves tissue perfusion and creates a more conducive environment for MSCs survival and function. Strategies to promote angiogenesis, such as activating the HIF-1 signaling pathway, supplementing copper, and combining EPCs and MSCs-based therapies, have shown promising results in preclinical studies. Future research should focus on elucidating the mechanisms underlying MSCs promotion of myocardial regeneration, developing novel techniques to rejuvenate the ischemic microenvironment, and conducting rigorous clinical trials to validate the efficacy and safety of these therapeutic approaches. With continued advancements in stem cell biology and regenerative medicine, MSCs-based therapies hold great potential for treating IHD, ultimately improving patient outcomes and quality of life.
